# Illustrating risk difference and number needed to treat from a randomized controlled trial of spinal manipulation for cervicogenic headache

**DOI:** 10.1186/1746-1340-18-9

**Published:** 2010-05-24

**Authors:** Mitchell Haas, Michael Schneider, Darcy Vavrek

**Affiliations:** 1 University of Western States, 2900 NE 132nd Avenue, Portland Oregon, USA; 2 University of Pittsburgh, 6035 Forbes Tower, Pittsburgh, Pennsylvania, USA

## Abstract

**Background:**

The number needed to treat (NNT) for one participant to benefit is considered a useful, clinically meaningful way of reporting binary outcomes from randomized trials. Analysis of continuous data from our randomized controlled trial has previously demonstrated a significant and clinically important difference favoring spinal manipulation over a light massage control.

**Methods:**

Eighty participants were randomized to receive spinal manipulation or a light massage control (n = 40/group). Improvements in cervicogenic headache pain (primary outcome), disability, and number in prior four weeks were dichotomized into binary outcomes at two thresholds: 30% representing minimal clinically important change and 50% representing clinical success. Groups were compared at 12 and 24-week follow-up using binomial regression (generalized linear models) to compute the adjusted risk difference (RD) between groups and number needed to treat (NNT) after adjusting for baseline differences between groups. Results were compared to logistic regression results.

**Results:**

For headache pain, clinically important improvement (30% or 50%) was more likely for spinal manipulation: adjusted RD = 17% to 27% and NNT = 3.8 to 5.8 (p = .005 to .028). Some statistically significant results favoring manipulation were found for headache disability and number.

**Conclusion:**

Spinal manipulation demonstrated a benefit in terms of a clinically important improvement of cervicogenic headache pain. The use of adjusted NNT is recommended; however, adjusted RD may be easier to interpret than NNT. The study demonstrated how results may depend on the threshold for dichotomizing variables into binary outcomes.

**Trial Registration:**

ClinicalTrials.gov NLM identifier NCT00246350.

## Background

The number needed to treat (NNT) for one participant to benefit from an intervention is considered a useful, clinically meaningful way of reporting binary outcomes from randomized trials [1,2]. It is the number of participants that must be treated for one clinical event to be attributable to a treatment above and beyond the benefit from a control. NNT is computed as one divided by risk difference (RD), where the RD (also known as absolute risk or absolute risk reduction), is the difference in proportions of participants achieving a clinical benefit in the treatment and control groups. For example, there is a RD of 20% when 60% of the participants in the treatment group achieve a clinical success as compared to 40% of participant s in the control group. In this case, the NNT would be 5.0.

Reporting NNT and RD has been recommended for randomized trials in the CONSORT statement [3], but these statistics have not been well utilized [4]. These measures can reveal important effects of care that are not reflected in the odds ratio, a statistic often reported for binary data [5,7].

Clearly, binary analysis is appropriate with naturally dichotomous variables, such as cure or death. When outcomes are evaluated as continuous variables, such as scales that measure pain intensity and functional disability, meaningful scale cut points must be identified to define the clinical result of interest. One reasonable cut point is the minimal clinically important difference or change to the participant. Ostello et al [8] reported the results of a literature review and expert panel. They concluded that a 30% improvement was a robust delineator of minimal important change across a number of pain and functional disability instruments. Thirty percent improvement was used in the UK BEAM trial for computing NNT [9]. Fritz et al [10] recommend using clinical success as an outcome, which they defined as 50% improvement in function disability for low back pain participants. The 50% improvement threshold is commonly reported in headache studies and we have previously reported this measure for headache pain and frequency [11].

We conducted a randomized trial evaluating the efficacy of spinal manipulation and comparing two doses of intervention provided by a chiropractor for the care of cervicogenic headache [11,13]. Spinal manipulation had a clinically important advantage over light massage in headache pain, number, and disability; there was little effect of dose. A path analysis suggested that a trial on manipulation can be designed where expectancy and the participant-provider encounter have minimal effect on outcomes [12]. Also, cervical pain-pressure thresholds may be determinants of clinical outcomes [13]. Our primary publication emphasized the differences between groups evaluated on continuous data scales, but did not give perspective on the proportion of participants expected to benefit from spinal manipulation [11].

The purpose of this article is to report clinician-friendly outcomes, RD and NNT for a minimal clinically important change (≥30% improvement) and successful treatment (≥ 50% improvement), and to discuss some advantages and shortcomings of these summary measures. Also useful in practice for formulating prognosis are charts estimating the probability of achieving different levels of improvement following treatment. For researchers, statistical methods are recommended for adjusting RD and NNT for baseline differences between treatment and control groups.

## Methods

### Design

The methods for this prospective randomized controlled trial are presented in detail in two previous publications [11,12]. Briefly, participants were randomized to receive either spinal manipulation or a minimal light massage control (n = 40 per group) provided by a chiropractor. Participants were further randomized to eight or 16 treatments over eight weeks. Treatment visits were 10 minutes in duration. Dose had little effect on outcomes in this study [11], and was therefore ignored in the analysis for this report. Randomization was conducted using computer-generated, design adaptive allocation to balance seven variables across groups (see *Statistical analysis*). Allocation was concealed from all personnel prior to randomization using this technique [11,12].

Data used in this report were collected at two baseline screening visits and by mailed questionnaire at 12 and 24 weeks. Missing data were imputed from outcomes collected through phone interview by a blinded research assistant at four, eight, 16, and 20 weeks. The primary outcome, identified in advance, was self-reported cervicogenic headache pain intensity. Analysis was conducted using the intention-to-treat principle. The trial was approved by the University of Western States Institutional Review Board (FWA 851).

### Participants

Volunteers were eligible if they had a history of at least 5 cervicogenic headaches per month for 3 months, with cervicogenic headache as defined by the International Headache Society in 1998 (excluding the radiographic criterion) [14]. Participants had a minimum score of 25 on the 100-point pain intensity scale to prevent floor effects. Participants were ineligible if they had contraindications to spinal manipulation [15], referred neck pain of organic origin, or pregnancy. Persons were also ineligible if they experienced other types of headache with etiologies that might have confounded the effects of manipulation on the cervicogenic component: cluster, metabolic/toxic, sinus, and headache associated with temporomandibular disease, tumors, and glaucoma [11,12].

### Assessment and intervention

A chiropractor/faculty member with 15 years experience screened volunteers for study eligibility through case history, standard orthopedic/neurological exam, heat sensitivity test, and 3-view cervical x-ray using the protocols of Vernon [16] and Souza [17] for cervicogenic headache and those of Gatterman and Panzer [15] for the cervical region. Four chiropractors with over 20 years of experience each served as the study treatment providers.

The treatment group received high velocity, low amplitude spinal manipulation of the cervical and upper thoracic (transitional region) spine at each visit as described by Peterson and Bergmann [18]. Modifications in manipulation recommended for older participants were permitted as required [19,20]. To relax the neck and upper back in preparation for spinal manipulation [21], the chiropractor administered a moist heat pack for five minutes and conducted a light massage for two minutes (described next).

The control group received five minutes of moist heat followed by five minutes of light massage. Light massage consisted of gentle effleurage (gliding) and gentle pétrissage (kneading) of the neck and shoulder muscles [22,23]. This allowed us to control contact with the participant with an intervention that was expected to have relatively small specific effects. This was because SMT had been shown to be superior to deep massage[24] and the LM application was much lighter and of much shorter duration than found in massage trials and common practice [25,26].

### Study variables for this report

Cervicogenic headache pain (CGH) intensity and disability were evaluated using the Modified Von Korff pain scale of Underwood et al [27]. The primary outcome was the pain scale and is the average of three 11-point numerical rating scales: CGH pain today, worst CGH pain in the last four weeks, and average CGH pain in the last four weeks. The disability scale (secondary outcome) is the average of three 11-point scales evaluating interference with daily activities, social and recreational activities, and the ability to work outside or around the house. The scales are scored from 0 to 100 with a lower score more favorable. The third outcome was the number of CGH in the previous four weeks. Baseline variables were used as covariates in the analysis. These included CGH pain and number, age, gender, self-reported previous diagnosis of migraine, confidence in care, and expected number of treatments needed for improvement. Treatment expectancy was evaluated with six-point Likert scales on participant confidence in the success of the two interventions using Interstudy's Low Back Pain TyPE Specification instrument [28].

### Statistical analysis

An intention-to-treat analysis was conducted with each participant included in the original allocation group with missing data imputed [11]. Five subjects were eliminated from this analysis due to lack of follow-up after baseline. For this secondary analysis, the continuous outcomes were dichotomized with 30% and 50% improvement as the threshold values for benefit and success, respectively.

Adjusted RDs between manipulation and control interventions were calculated using a test of proportions called binomial regression (a generalized linear regression model) that takes into account differences between groups in baseline covariates [29,30]. The covariates for all analyses are listed under *Study variables *above. Multiple logistic regression was first performed to calculate an initial estimate of the mean of the dependent variable for the binomial regression analysis. When a binomial regression model failed to converge and yield an estimate of the RD, multiple linear regression was used to estimate the RD between groups [31,32]. All analyses used robust standard errors to minimize distributional assumptions [31]. The adjusted NNT and 95% confidence intervals were then computed by inverting the adjusted RD and its 95% confidence limits. Logistic regression was also used to compute odds ratios comparing interventions adjusted for the baseline covariates [29]. All analyses were conducted with Stata 11 (Stata Corp, College Station, TX).

## Results

**Figure 1:**
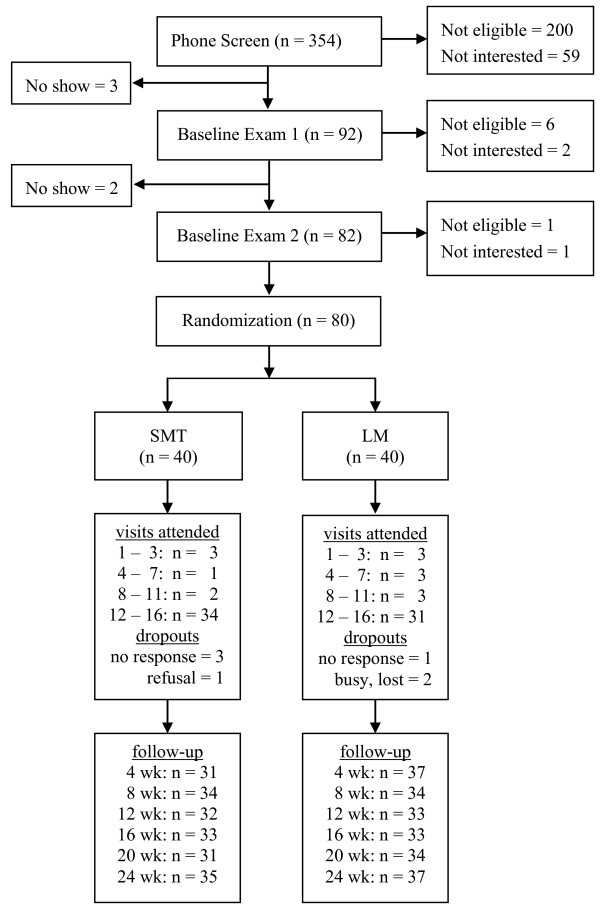
**Study flowchart**.

The study flow chart with details of adherence to treatment and compliance with follow-up are presented in Figure 1 with further details published elsewhere [11]. Participant adherence to study visits was 86% on average. Compliance with follow-up was 83% and 90% at 12 and 24 weeks, respectively. Baseline characteristics are presented in Table 1. Participants were generally young and predominantly women. They averaged about four cervicogenic headaches per week and had a mean headache pain intensity of 54.3 (SD = 16.9) and mean disability of 45.0 (SD = 22.9). About a quarter were also migraine sufferers. Differences between groups were noted for headache pain and disability.

**Table 1:** Baseline participant characteristics*

	SMT (n = 40)	LM (n = 40)	All (n = 80)
Sociodemographic information			
Age (years)	37 ± 11	36 ± 11	36 ± 11
Gender (female)	80%	80%	80%
Cervicogenic headache			
Pain intensity (100-point scale)	50.9 ± 17.0	57.8 ± 16.3	54.3 ± 16.9
Number of headaches in the past 4 weeks	15.4 ± 8.0	16.0 ± 7.8	15.7 ± 7.9
Disability (100-point scale)	42.7 ± 23.1	47.4 ± 22.9	45.0 ± 22.9
Migraine sufferer (self report)	30%	25%	28%
Optimal number of treatments (4 - 20)			
SMT	12.9 ± 4.9	12.5 ± 5.1	12.7 ± 5.0
LM	14.3 ± 4.8	14.2 ± 5.4	14.3 ± 5.0
Expectations †			
Confidence in success of SMT	4.2 ± 1.2	4.1 ± 1.3	4.1 ± 1.3
Confidence in success of LM	4.0 ± 1.2	4.1 ± 1.2	4.1 ± 1.2

SMT - spinal manipulative therapy; LM - light massage.

* Values are means ± SD or percentage.

† 6-point Likert scale anchored by 1 = extremely uncertain and 6 = extremely certain.

### Cervicogenic headache pain

Table 2 shows the observed percentage of participants achieving improvement in the spinal manipulation and control groups, as well as the adjusted RD and adjusted NNT. A substantial percentage of participants achieved the 30% and 50% thresholds for improvement at 12 and 24 weeks after randomization. The difference between treatment and control groups strongly favored spinal manipulation over the light massage control after correcting for baseline differences between groups (P = .005 to .028). The NNT was about four; that is, only four participants required treatment for one participant to benefit from manipulation itself, above that which was achieved by light massage.

**Table 2:** Participants obtaining 30% and 50% improvement in outcomes: risk difference (RD) and number needed to treat (NNT)*

Improvement criterion		SMT (n = 36)	LM (n = 39)	adjusted RD (95% CI)	P	adjusted NNT (95% CI worst to best)
Cervicogenic headache pain scale †						
30%	12 wk	47%	38%	20% (2, 37)	.025	5.1 (40, 2.7)
	24 wk	58%	49%	17% (2, 33)	.028	5.8 (54, 3.1)
50%	12 wk	42%	23%	26% (8, 45)	.005	3.8 (13, 2.2)
	24 wk	42%	23%	27% (8, 46)	.006	3.8 (13, 2.2)
Cervicogenic headache number (in last 4 wk)						
30%	12 wk	72%	51%	23% (6, 40)	.007	4.3 (16, 2.5)
	24 wk	78%	62%	14% (1, 27)	.040	7.2 (152, 3.7)
50%	12 wk	64%	46%	21% (0, 43)	.048	4.7 (442, 2.4)
	24 wk	61%	51%	14% (-2, 30)	.094	7.2 (-42, ± ∞, 3.3)
Cervicogenic headache disability scale †						
30%	12 wk	64%	51%	15% (-9, 39) ‡	.226	6.7 (-3.5, ± ∞, 2.6)
	24 wk	61%	64%	-7% (-29, 15)	.553	-15 (-11, ± ∞, 6.5)
50%	12 wk	64%	36%	29% (6, 52) ‡	.015	3.5 (17, 1.9)
	24 wk	56%	38%	20% (-1, 42)	.061	4.9 (-105, ± ∞, 2.4)

SMT - spinal manipulative therapy; LM - light massage; NNT - number needed to treat

* Outcomes are presented for the 12-week (short-term) and 24-week (intermediate-term) follow-ups. The SMT and LM group percentages are unadjusted. Missing data were imputed except for five participants with no follow-up data. Differences between groups (risk differences) were adjusted for baseline and all randomization variables. Adjusted NNT = one divided by the adjusted difference between groups. Positive numbers favor spinal manipulation. For the NNT CIs, the limit most favorable to manipulation is on the right and least favorable on the left. Note that for statistically insignificant results, the RD confidence interval includes zero, so that the NNT confidence interval must include 1/0 = ± ∞. These infinity values are more favorable to SMT than a small negative number and less favorable than a small positive number.

† Modified Von Korff scale (scored from 0 to 100 points before dichotomization).

‡ Linear least-squares regression used in place of binomial regression.

Table 2 can be interpreted as follows, using the third row of data as an example (participants achieving 50% improvement at 12 weeks): Successful outcomes were achieved in 42% of SMT participants and 23% of control participants. When correcting for baseline differences between the two study groups, the adjusted RD = 26%. This means that we can estimate that 26% or about one in four participants are expected to have a successful treatment outcome that is directly attributable to SMT above the success rate that we can expect from a minimal light massage. The confidence interval for the adjusted RD was 7.9% to 45%. This tells us that the true RD for the study population may be considerably lower or higher than 26%; this is a consequence of the modest sample size. The statistical significance is P = .005. It suggests that the favorable results for SMT are unlikely due to chance (sampling error) alone. The p-value applies to both the adjusted RD and NNT. Putting it all together, we can conclude that an advantage for SMT is likely real (P = .005) and substantial (adjusted RD = 26%), but the advantage can range with equal probability from the small (lower 95% confidence limit = 7.9%) to the extremely large (upper 95% confidence limit = 45%).

The adjusted NNT is, in essence, a rewording of the adjusted RD. It shows that we expect to treat 3.8 participants for one participant to benefit from SMT over the control intervention (i.e., about one in four participants benefit directly). The confidence interval shows that the NNT in the true study population is likely between a mediocre 13 and an extremely favorable two. Note that in general, NNT of large magnitude indicate trivial differences between interventions and small NNT indicate large differences. One is the smallest possible NNT and occurs in the extremely unlikely case of a 100% success in the treatment group and 0% success in the control group.

### Cervicogenic headache number

Reduction in the monthly number of headaches also favored SMT (Table 2). At 12 weeks, the adjusted RD was 21% to 23% and the adjusted NNT was 4.3 to 4.7 participants. At 24 weeks, the adjusted RD was 14% and the adjusted NNT was 7.2. The results were statistically significant (P < .05) except for 50% improvement at the 24-week follow-up (P = .094).

Note that for results that are not statistically significant, one side of the 95% confidence interval (CI) will be negative for both RD and NNT. The CI can be interpreted moving from the left limit to the right limit of the CIs for 50% improvement at 24 weeks in Table 2. For RD, the CI is -2 to 30. The left limit is -2, indicating a possible advantage for the control group; the CI passes zero (the break-even point); and the upper limit is 30, indicating the largest advantage for the treatment group in the CI. The CI for NNT (-42, ± ∞, 3.3) is more complicated. It starts with the smallest negative number on the left (-42), which indicates the largest advantage for the control group. Moving toward the center of the CI, the negative value increases to negative infinity (NNT = 1/RD = -1/0 = -∞), the smallest advantage for the control (none). As the break-even point is crossed, the value flips to positive infinity (+1/0 = +∞), the smallest advantage for SMT (none). The positive number decreases in size until the upper limit is reached (3.3), the largest advantage for the treatment group.

### Cervicogenic headache disability

Results were mixed for headache disability (Table 2). Outcomes were favorable for manipulation at 50% improvement albeit marginally failing to reach statistical significant at 24 weeks (NNT = 3.5, P = .015 and NNT = 4.9, P = .061). There were no significant outcomes for 30% improvement.

### Risk differences and odds ratios

Table 3 shows the traditionally reported adjusted odds ratios comparing study groups. A comparison of Table 2 and Table 3 show that statistical significance for RD and odds ratios were not consistent for all comparisons between manipulation and control. There were four statistically significant results for the odds ratio and four additional statistically significant findings using binomial regression.

**Table 3:** Participants obtaining 30% and 50% improvement in outcomes: odds ratio*

Improvement criterion		SMT (n = 36)	LM (n = 39)	adjusted odds ratio (95% CI)	P
Cervicogenic headache pain scale †					
30%	12 wk	47%	38%	2.2 (0.8, 6.0)	.142
	24 wk	58%	49%	2.3 (0.8, 6.5)	.122
50%	12 wk	42%	23%	3.0 (1.1, 8.3)	.033
	24 wk	42%	23%	3.6 (1.3, 9.7)	.011
Cervicogenic headache number (in last 4 wk)					
30%	12 wk	72%	51%	4.4 (1.3, 14.3)	.015
	24 wk	78%	62%	3.1 (0.9, 11.2)	.082
50%	12 wk	64%	46%	2.6 (0.9, 7.5)	.067
	24 wk	61%	51%	1.9 (0.7, 5.2)	.225
Cervicogenic headache disability scale †					
30%	12 wk	64%	51%	2.0 (0.7, 5.7)	.193
	24 wk	61%	64%	0.9 (0.3, 2.4)	.803
50%	12 wk	64%	36%	3.8 (1.3, 11.0)	.014
	24 wk	56%	38%	2.2 (0.9, 5.9)	.102

SMT - spinal manipulative therapy; LM - light massage; NNT - number needed to treat

* Outcomes are presented for the 12-week (short-term) and 24-week (intermediate-term) follow-ups. The SMT and LM group percentages are unadjusted. Missing data were imputed except for five participants with no follow-up data. Odds ratios were adjusted for differences between groups in the baseline value of the outcome and all randomization variables. Ratios greater than 1.0 favor spinal manipulation.

† Modified Von Korff scale (scored from 0 to 100 points before dichotomization).

### Expected improvement in practice

**Figure 2:**
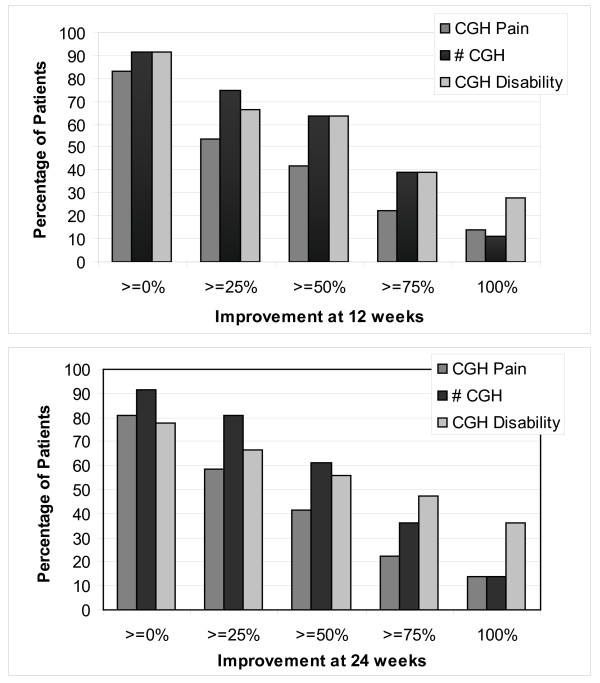
**Improvement from spinal manipulation**. The figures show the percentage of spinal manipulation patients that achieved increasing levels of improvement in cervicogenic headache (CGH) pain, number, and disability at the 12-week and 24-week follow-ups. Control group data are not included.

Figure 2 was designed as a pragmatic tool for estimating the probability of improvement for the treatment of cervicogenic headache with spinal manipulation. It shows the percentage of participants that achieved ≥ 0%, ≥25%, ≥75%, and 100% improvement at 12 and 24 weeks in the study. Quartiles were chosen for the convenience of creating easily readable bar graphs for the three study outcomes. Figure 2 shows that the percent improvement in disability and number of headaches was greater than the improvement in pain, although manipulation outperformed the control more for pain (Table 2). Outcomes at 12 weeks were durable to 24 weeks. About 40% to 60% of participants achieved a success threshold of 50% improvement and at least 10% had complete relief for at least one of the three outcomes. It should also be noted that about 10% to 20% reported poorer scores than at baseline at one of the follow-up time points, and there is room for improvement.

## Discussion

The analysis of percent improvement shows a benefit of spinal manipulation compared to a minimal light massage control at 12 and 24 weeks for the relief of cervicogenic headache pain. The evidence is not as consistent for the other outcomes, but some positive results were observed for headache number particularly in the short term.

The evidence-based practice movement favors the use of the NNT [33]; this requires dichotomizing continuous outcomes using a pre-determined threshold of benefit or success. Percent improvement thresholds yield complementary information to continuous scale data for interpreting a clinical outcome. For example, a 20-point improvement (on a 100-point scale) may be a large or small percentage depending on the baseline starting point. Alternatively, a 20-point improvement may be clinically important regardless of whether the improvement threshold criterion is met. Also, a 50% improvement may be clinically important despite the magnitude of change in the outcome score. It should be noted that a shortcoming of threshold percent improvement is that it is ultimately somewhat arbitrary. Table 2 gives a cautionary tale showing how the choice of a 30% or 50% threshold for treatment success can lead to different results in terms of RD, NNT, and statistical significance. This in turn can impact conclusions on the clinical importance of the target intervention.

It is our opinion that the RD is preferable to the NNT as an outcome measure because of the difficulty in interpreting the 95% CI of the NNT. If RD = 10% (95% CI = -5%, 20%), then we can say that one in 10 participants treated will have successful outcomes attributable to the treatment compared to the control. This is equivalent to saying it will take treating 10 participants to get one better (NNT = 10). The 95% confidence interval for the RD can be expressed as between one in 20 favoring the control (-5%) to one in four favoring the treatment (20%). The advantage of using the RD is that the confidence interval is easily interpretable: a small benefit favoring the comparison intervention to a sizable advantage for the index treatment. A value of zero clearly shows no difference between groups. There is no need to confront the perplexing 95% confidence interval of the NNT: 1) the inclusion of ± ∞ when results are not statistically significant and 2) confidence limits that get smaller in magnitude the further away they are from the null.

Expected improvement graphs (Figure 2) can be a most useful tool for the practicing clinician. Most importantly, they can be used for prognosis. Both participants and clinicians can see the chance of achieving different levels of improvement and form realistic expectations of treatment outcomes. The graphs are also easier to interpret than a table of means and standard deviations. The improvement rates for the control group could be included to add the perspective of improvement relative to a sham, no intervention, or other therapy. We did not include the control group in our graphs for ease of interpretation.

### Technical notes on analysis

Adjusted RD and adjusted NNT are recommended to take into consideration baseline differences between groups on important predictors of outcomes. This is especially important in small studies where imbalances in baseline characteristics are more likely to occur. Binomial regression is a generalized linear model that can compute differences between groups after adjusting for baseline covariates when the dependent variable (outcome) is a proportion; it assumes a binomial distribution for the outcome measure [29,30]. The shortcoming of binomial regression is that it uses an iterative algorithm that must converge to an RD estimate. Often the model fails to converge or gives a poor estimate of the RD [31]. One way to get around this is to run a logistic regression model to give an estimate of risk (probability of improvement in our study) for each individual. This risk can then be used to pre-specify the initial estimate of the mean for the dependent variable for the iterative process in binomial regression. This method was used for all our binomial regression analyses, because many models failed to converge without an initial estimate of the mean. In the two cases where the models still failed to converge, the following analysis was conducted.

An alternative analysis to binomial regression is ordinary least-squares multiple linear regression with the dependent variable coded as zero or one for the two values of the dichotomized outcome [31,32]; this is also called modified least-squares regression when a robust standard error is used [31]. The usual estimate of the difference between group means (grouping variable regression coefficient) turns out to be an estimate of the difference between group proportions as in binomial regression.

Odds ratios from logistic regression are commonly reported in epidemiological studies. In randomized trials, logistic and binomial regression can give different perspectives on outcomes. For example, consider two experiments were the RD = 40% - 20% = 20% in one and 10% - 5% = 5% in the other. The RD of the first is four times that of the second, but the odds ratios are similar, 2.67 and 2.1, respectively [5]. Binomial regression is more difficult to use because of the convergence problem [31], but logistic regression cannot be readily used to compute the NNT.

## Conclusion

The use of RD and NNT adjusted for baseline differences between groups in important determinants of outcomes is recommended for randomized trials with binary or dichotomized outcomes. The RD and its derivative the NNT are more clinician friendly than the odds ratios, and the RD in particular has an easier confidence interval to interpret than the NNT. Tabulation of the expected percentage of participants with successful care is a practical tool for the clinician.

Specifically in our study, spinal manipulation demonstrated a benefit in terms of a clinically important improvement of cervicogenic headache pain compared to a control when using a 30% and a 50% threshold for defining improvement. Our study demonstrated how results and interpretation may vary depending on the threshold chosen for dichotomizing continuous variables into binary outcomes.

## Competing interests

The authors declare that they have no competing interests.

## Authors' contributions

MH was responsible for the experimental design, implementation of the randomized trial, analysis and interpretation of the data, and manuscript preparation.

MS and DV conducted data analysis and participated in data interpretation and manuscript preparation.

All authors have read and approved the final manuscript.
